# Age- and Sex-Specific Gut Microbiota Signatures Associated with Dementia-Related Brain Pathologies: An LEfSe-Based Metagenomic Study

**DOI:** 10.3390/brainsci15060611

**Published:** 2025-06-05

**Authors:** Sun Hwa Hong, Hyun Woong Roh, You Jin Nam, Tae Wi Kim, Yong Hyuk Cho, Sang Joon Son, Chang Hyung Hong

**Affiliations:** Department of Psychiatry, Ajou University School of Medicine, Suwon 16499, Republic of Korea; sh9@ajou.ac.kr (S.H.H.); hansin8607@ajou.ac.kr (H.W.R.); yjnam@ajou.ac.kr (Y.J.N.); rlaxodnl@ajou.ac.kr (T.W.K.); brainist.cho@gmail.com (Y.H.C.); sjsonpsy@gmail.com (S.J.S.)

**Keywords:** gut microbiota, dementia-related brain pathologies, sex-specific differences, age-related characteristics, metagenomic analysis, biomarkers, *Bifidobacterium* spp., *Bacteroides* spp., microbial diversity, personalized therapy

## Abstract

Background/Objectives: Emerging evidence suggests that gut microbiota composition is influenced by both age and sex and may contribute to dementia-related brain pathologies. However, comprehensive microbiome-based biomarker discovery stratified by these factors remains limited. Methods: We performed a metagenomic analysis of the gut microbiota of participants stratified by sex (female vs. male) and age (<75 vs. ≥75 years). Alpha diversity (observed operational taxonomic unit, Chao1, Shannon, and Simpson) and linear discriminant analysis effect size analyses were conducted to identify dominant taxa associated with Alzheimer’s pathology, vascular pathology, and dementia-related structural brain changes. Results: Females and non-elderly participants (aged < 75 years) exhibited higher gut microbial diversity, characterized by an increased abundance of *Bifidobacterium* spp. and *Blautia* spp., whereas males and elderly participants (aged ≥ 75 years) exhibited increased levels of *Bacteroides* spp. and *Bacteroidia*, which have been associated with inflammation and dysbiosis. Several taxa, including *Bifidobacterium* spp. were consistently identified as potential protective biomarkers, while *Bacteroides* spp. was linked to a higher risk of dementia-related brain pathologies. Conclusions: Our findings demonstrate distinct age- and sex-specific differences in gut microbiota composition that may be closely associated with the pathophysiology of dementia-related brain pathologies. These results demonstrate that gut microbiota may serve as potential biomarkers for monitoring cerebrovascular conditions, potentially contributing to the development of personalized therapeutic strategies.

## 1. Introduction

The global population is aging at an unprecedented rate, with a rapidly increasing proportion of older adults. As of 2024, individuals aged 65 years and older account for approximately 10.2% of the global population, and this proportion is projected to rise to 20.3% by 2072 [1]. Similarly, the United Nations’ World Population Prospects 2024 report [2] estimates that the proportion of individuals aged 65 or older will increase from 10.0% in 2023 to 23.9% by 2100. This demographic trend accordingly highlights the urgent need for multidisciplinary strategies to address the medical, social, and economic challenges associated with population aging.

Aging has been identified as a major risk factor for neurodegenerative diseases. In this regard, Hou et al. have reported that aging-related cellular and molecular changes play key roles in the pathogenesis of dementia, Alzheimer’s disease, Parkinson’s disease, and dementia-related brain pathologies via a diverse range of mechanisms [3]. Among the consequences of aging, the increasing burden of dementia-related brain pathologies, particularly dementia, poses significant social and economic challenges on account of the need for long-term care, rising healthcare expenditures, and loss of productivity among family caregivers [4,5,6]. Nichols et al., who conducted a systematic analysis of the global burden of Alzheimer’s disease and other dementias between 1990 and 2016, have reported a 148% increase in dementia-related mortality and a substantial rise in disability-adjusted life years (DALYs), which contribute to exacerbating the economic impact on aging populations [6]. Likewise, Livingston et al. have highlighted the growing social and economic burden of dementia and emphasized the importance of early interventions and multidisciplinary approaches to mitigate the impact of aging on the prevalence of dementia [4].

Dementia often remains asymptomatic for years or even decades, and by the time clinical symptoms manifest, significant neuropathological damage may have already occurred [7]. Traditional diagnostic approaches, which are primarily dependent on cognitive and clinical assessments, are often limited to identifying the disease in its later stages. Thus, there is a growing demand for systems with the capacity to detect early pathological changes and thereby enable timely interventions. In this regard, biomarker-based diagnostic strategies have emerged as promising tools for early detection, moving beyond symptom-based diagnosis [8,9,10]. The authors of these studies have proposed novel approaches for enhancing predictive accuracy and standardization in dementia research, including non-invasive blood-based diagnostics using plasma P-tau181 [9], phospho-tau217 for distinguishing Alzheimer’s from other neurodegenerative diseases [10], and combinations of specific proteins and inflammatory markers for early-stage detection. Additionally, MRI and PET imaging [11] and cerebrospinal fluid (CSF) biomarkers [12,13] have shown value in predicting disease progression. Beyond traditional biomarkers, recent studies have highlighted the potential role of the gut microbiota in brain health and disease. In their meta-analysis on the interactions between the gut microbiome and brain function, Cryan et al. introduce the concept of the microbiota–gut–brain axis [14]. Their findings indicate that alterations in the gut microbiota are linked to neurodevelopmental and neurodegenerative disorders and may serve as potential biomarkers for brain diseases. Furthermore, Sharon et al. have examined the associations between the gut microbiota and the central nervous system (CNS), reporting correlations with autism spectrum disorders, multiple sclerosis, and Parkinson’s disease [15]. These studies accordingly serve to emphasize that the gut microbiome may serve not only as a source of biomarkers but also provide therapeutic targets. Recent evidence increasingly supports that alterations in gut microbiota composition play a pivotal role in the pathogenesis of Alzheimer’s disease and other dementia-related disorders by modulating neuroinflammation, immune responses, and the gut–brain axis [16]. However, despite these advances, age- and sex-specific gut microbiome profiles associated with Alzheimer’s pathology, vascular pathology, and dementia-related structural brain changes remain poorly characterized, underscoring the need for further investigation. Furthermore, experimental studies have demonstrated that modulation of the gut microbiota can influence neurodegenerative disease progression. For example, Sampson et al. reported that modifying the gut microbiota alleviated motor symptoms and neuroinflammation in a Parkinson’s disease mouse model, reinforcing its potential as a predictive and diagnostic biomarker [17]. Similarly, fecal microbiota transplantation (FMT) experiments in animal models have shown that gut microbiome alterations can affect the pathology and progression of Alzheimer’s disease [18]. Collectively, these findings support the hypothesis that the gut microbiota plays a critical role in the onset and progression of neurodegenerative diseases and may serve as a promising target for diagnostic and therapeutic interventions through the gut–brain axis. This study aimed to characterize age- and sex-specific gut microbiota profiles associated with dementia-related brain pathologies. Our goal was to identify potential microbial biomarkers that could contribute to the early detection and risk stratification of dementia-related brain pathologies and dementia.

## 2. Materials and Methods

### 2.1. Sample Collection

This study was conducted as part of the Biobank Innovations for Chronic Cerebrovascular Disease with Alzheimer’s Disease Study (BICWALZS), a nationwide initiative led by the Korea Disease Control and Prevention Agency. Launched in 2016 through a collaboration between Ajou University School of Medicine and the National Biobank of Korea, BICWALZS aims to facilitate the efficient use of human biospecimens and real-world clinical data derived from individuals with subjective cognitive decline, mild cognitive impairment, Alzheimer’s disease, and subcortical vascular dementia.

The study protocol was approved by the Institutional Review Board of Ajou University Hospital, Suwon, Republic of Korea (approval number: AJOUIRB-SUR-2021-038), and written informed consent was obtained from all participants and their legal guardians prior to enrollment. A total of 292 participants recruited from the Memory Clinic at Ajou University Hospital and the Suwon Mental Health Center for the Elderly, Gyeonggi-do, Republic of Korea, were included in this study. All participants were East Asian individuals of Korean descent. Stool samples were collected and banked for microbiome analysis. To ensure diagnostic accuracy, each participant underwent structural brain MRI and amyloid PET imaging. In addition, baseline clinical data and blood samples were collected for biochemical and cognitive assessments.

Participants were diagnosed based on a comprehensive assessment including clinical interviews, laboratory tests, and standardized neuropsychological evaluations. Clinical diagnoses of subjective cognitive decline (SCD), mild cognitive impairment (MCI), Alzheimer’s disease dementia (AD), and vascular dementia (VD) were made according to established international criteria. In addition to clinical evaluation, multimodal biomarker assessments such as brain magnetic resonance imaging (MRI), amyloid positron emission tomography (PET), and genetic analyses (single nucleotide polymorphism microarray) were incorporated to enhance diagnostic precision [19].

### 2.2. Gut Microbiota Analysis

#### 2.2.1. Bacterial DNA Extraction

Human fecal samples were mixed with 6 mL of phosphate-buffered saline (PBS) and left to sit for 24 h, after which the mixture was filtered through a cell strainer. The filtered solution was centrifuged at 10,000× *g* for 10 min at 4 °C. Following this step, the bacterial pellet was carefully separated. The bacterial pellet was resuspended in 200 μL of PBS, and the supernatant was filtered using a 0.2 μm syringe filter and then heated to 100 °C for 40 min using a heat block. Residual particles and debris were eliminated via centrifugation at 18,312× *g* for 30 min at 4 °C. DNA was extracted from the bacterial fraction using a DNeasy PowerSoil Pro Kit (Qiagen, Hilden, Germany), following the manufacturer’s instructions. The extracted DNA was quantified using a QIAxpert system (Qiagen, Hilden, Germany).

#### 2.2.2. Analysis of Microbial Community Composition

Adapter sequences from paired-end reads were removed using Cutadapt (v1.1.6) [20]. The cleaned reads were then merged using CASPER, and only high-quality sequences, as defined by Phred score filtering criteria (Q score) from previous studies [21,22], were retained. Sequences outside the size range of 350 to 550 bp after merging were excluded from further analysis. Chimeric sequences were identified through reference-based detection using VSEARCH (v2.0.3.), aligned against the SILVA (v138) gold database [23,24]. For taxonomic classification, the quality-filtered sequences were clustered into operational taxonomic units (OTUs) using a closed-reference method with 97% sequence identity, also via VSEARCH. Representative OTU sequences were annotated using the SILVA 138 database through the UCLUST classifier implemented in QIIME (v1.9.1) with default settings [25]. To evaluate alpha diversity, the Chao1 richness estimator was calculated for each sample. If genus-level classification was not possible due to limitations in the sequence data or reference database redundancy, taxonomy was assigned to the next highest reliable taxonomic level, as indicated in parentheses.

### 2.3. Statistical Analysis

Multivariate logistic regression analyses were performed to identify potential risk factors associated with Alzheimer’s pathology (negative/positive), vascular pathology (negative/positive), and dementia-related structural brain changes (0 = mild, 1 = moderate to severe). Each brain pathology was used as a dependent variable in separate models, while the independent variables included sex (female/male), age (continuous), presence of Alzheimer’s pathology (negative/positive), presence of vascular pathology (negative/positive), and degree of dementia-related brain changes (0 = mild, 1 = moderate to severe). The results are presented as odds ratios (ORs) with corresponding 95% confidence intervals. Statistical significance was set at a *p*-value < 0.05. All analyses were conducted using SPSS software (version 29.0K; SPSS Inc., Chicago, IL, USA). The assumptions of logistic regression (e.g., absence of multicollinearity and linearity in the logit) were assessed and satisfied prior to analysis. As logistic regression does not require normality assumptions, only linearity in the logit was assessed for continuous variables and confirmed prior to analysis.

Linear discriminant analysis effect size (LEfSe) was applied to identify significantly discriminative taxa between groups. The threshold for logarithmic LDA score was set at 2.0 to define dominant taxa, and statistical significance was determined using the default LEfSe algorithm parameters.

## 3. Results

### 3.1. Distribution of Brain Pathologies by Age and Sex

We investigated the influence of age and sex on the distribution of dementia-related brain pathologies (Table 1). Alzheimer’s pathology, vascular pathology, and dementia-related structural brain changes were classified based on the presence of Alzheimer’s pathology, vascular pathology, and dementia-related structural brain changes. These classifications were determined through expert clinical evaluation, incorporating findings from brain MRI and amyloid PET scans. 

In this study, we examined the distribution of three distinct dementia-related brain pathologies across age and sex: Alzheimer’s pathology, vascular pathology, and dementia-related structural brain changes. Although these pathologies often co-occur in aging individuals, they are biologically and mechanistically distinct.

Alzheimer’s disease (AD) is characterized by progressive neurodegeneration driven by amyloid-β plaque deposition and neurofibrillary tangles composed of hyperphosphorylated tau protein. In contrast, vascular pathology (VP) is the result of cerebrovascular dysfunction such as ischemia, microinfarcts, or hemorrhagic lesions leading to structural and functional impairment of brain tissue. Meanwhile, dementia-related structural brain changes refer to imaging-based evidence of global or regional atrophy and white matter changes, which may arise from either or both AD and VP but were independently classified in this study.

We evaluated the prevalence of each pathology among 292 participants stratified by age and sex, as shown in Table 1. Notably, the proportion of participants positive for Alzheimer’s pathology increased with age, with the highest frequency observed in females aged ≥75 years (10.3%) and 60–74 years (8.6%). A similar age-related increase was observed for vascular pathology, which was most prevalent in elderly females (14.0%). In younger participants aged 40–59 years, the prevalence of both pathologies remained low (<1%).

Dementia related structural changes defined as moderate to severe abnormalities on neuroimaging also showed a significant age-related trend. The highest rates were found in females aged ≥ 75 years (21.2%), followed by females and males aged 60–74 years. In contrast, no moderate to severe structural changes were observed in males aged 40–59 years.

These findings suggest that advancing age is a strong risk factor for the development of both AD and CVD, while sex-related patterns may influence vulnerability particularly among elderly females for AD, and elderly males for severe structural deterioration, as further analyzed in the following section. The clear separation in pathological mechanisms justifies the need for independent evaluation and biomarker profiling of each condition, despite their frequent co-occurrence in older adults.

### 3.2. Predicted Risk of Alzheimer’s Pathology, Vascular Pathology, and Dementia-Related Structural Brain Changes According to Age and Sex

Table 2 presents the results of multivariate logistic regression analyses used to evaluate the influence of demographic variables, specifically age and sex, on the risk of Alzheimer’s pathology, vascular pathology, and dementia-related structural brain changes. The dependent variables were as follows: presence of Alzheimer’s pathology (positive), vascular pathology (positive), and moderate to severe dementia-related brain changes. The analysis revealed that older age was significantly associated with a higher risk of presenting Alzheimer’s pathology. Similarly, the likelihood of vascular pathology increased significantly with older age. Older age was also significantly associated with a higher probability of presenting moderate to severe brain changes. Furthermore, male sex was identified as a significant risk factor for developing moderate to severe dementia-related brain changes, indicating that males were more likely to exhibit such changes than females. These findings highlight the strong influence of age on all three Alzheimer’s pathology, vascular pathology, and dementia-related structural brain changes and suggest that sex-specific differences, particularly among older individuals, contribute to differential vulnerability to neurodegenerative brain changes.

Statistical analyses were conducted to identify significant risk factors associated with the development of moderate to severe dementia-related brain changes (Table 3). A multivariate logistic regression model was applied. The dependent variable was dementia-related brain changes (moderate to severe), and the independent variables were sex, age, presence of Alzheimer’s pathology, and presence of vascular pathology. The results demonstrated that male sex was significantly associated with a higher likelihood of exhibiting moderate to severe dementia-related brain changes (OR ≈ 4.24, *p* < 0.001). Older age was also a significant risk factor, with the odds increasing by approximately 1.09 times for each additional year of age (OR ≈ 1.09, *p* < 0.001). Additionally, the presence of vascular pathology was independently associated with an increased risk of developing moderate to severe dementia-related brain changes (OR ≈ 3.55, *p* < 0.001). These findings suggest that older males with vascular pathology are at a significantly higher risk of presenting advanced dementia-related brain changes. Furthermore, the data highlight the strong and independent effect of age as a continuous risk factor for dementia progression. In contrast, Alzheimer’s pathology was not a significant predictor in this model (*p* = 0.316), possibly due to the relatively stronger influence of vascular pathology, which may have been a more dominant independent variable in predicting structural brain deterioration.

### 3.3. Age-Related Changes in Gut Microbial Community Composition

**Figure 1:**
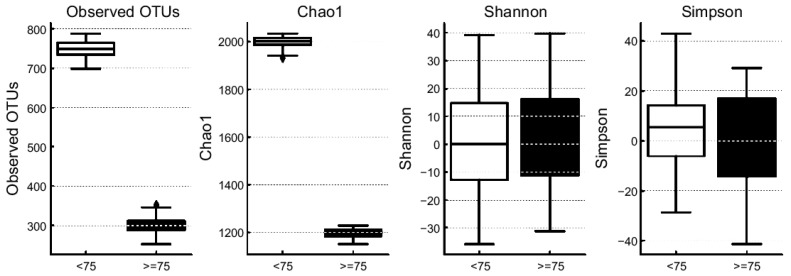
Comparison of alpha diversity metrics (observed OTUs, Chao1, Shannon, and Simpson) between <75-year-old and ≥75-year-old age groups. The <75-year-old group is shown in white and the ≥75-year-old group is shown in black. All diversity indices were significantly higher in the <75-year-old group (*p* < 0.05, Wilcoxon rank-sum test).

Aging significantly influences the structure and diversity of the gut microbiota, which is closely linked to metabolic disorders, inflammatory diseases, and cognitive decline. In this study, we evaluated the effect of aging on gut microbial ecology by stratifying participants into two age groups based on a cutoff of 75 years and comparing the gut microbiota composition between these groups (Figure 1).

Species richness within the gut microbiota was estimated using the Chao1 index. After rarefaction, a comparison of richness between the two age groups (<75 years vs. ≥75 years) revealed that the younger group exhibited higher richness than the older group (Figure 1). This suggests that individuals below the age of 75 years harbor a more diverse range of microbial species within their gut ecosystem. OTUs were clustered to count species-level microbial taxa, and rare OTUs (those observed only once or twice) were incorporated into Chao1-based estimates to adjust for unseen species and accurately assess richness. In addition to richness, Shannon and Simpson indices were used to evaluate microbial diversity and dominance patterns. The results showed a significant decline in microbial diversity in the ≥75-year-old group compared with the <75-year-old group. Specifically, the <75 group demonstrated higher observed OTUs and Chao1 richness, indicating a greater number of observed species as well as rare taxa. This indicated that microbial richness tended to decrease with older age. This age group also had a higher Shannon index, indicating greater diversity and evenness within the microbial community. Meanwhile, the lower Simpson index in this age group suggested a less dominant influence by a few species, reflecting a more evenly distributed microbial population structure.

**Figure 2:**
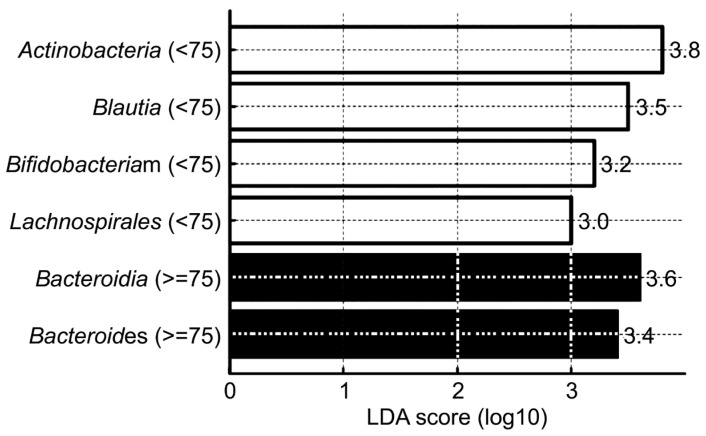
LEfSe-style linear discriminant analysis (LDA) scores showing dominant gut microbiota taxa by age group.

The gut microbiota from all 292 participants were analyzed using 16S rRNA-based next-generation sequencing. Taxonomic composition was characterized from the phylum to the species level to identify dominant microbial taxa. To assess significant differences in microbial composition between the two age groups, linear discriminant analysis (LDA) effect size (LEfSe) and non-parametric Wilcoxon rank-sum tests were conducted (Table 4, Figure 2).

A taxonomic comparison of gut microbial communities from phylum to species level revealed compositional differences between the two age groups (<75 years vs. ≥75 years). At the phylum level, *Actinobacteriota* was significantly more abundant in the <75 group (*p* = 0.004) while *Bacteroidota* was more abundant in the ≥75 group (*p* = 0.005). At the class level, *Actinobacteria* was relatively more prevalent in the <75 group (*p* = 0.0005) whereas *Bacteroidia* was significantly enriched in the ≥75 group (*p* = 0.005). At the order level, *Lachnospirales* was significantly more abundant in the <75 group (*p* = 0.00006), suggesting that Firmicutes-related bacterial taxa were better maintained in the younger population. At the genus level, *Bifidobacterium* and *Blautia* were significantly more abundant in the <75 group whereas *Bacteroides* showed higher relative abundance in the ≥75 group. These findings suggest that aging is associated with a restructuring of the gut microbiome, characterized by a shift toward dominance of specific taxa such as *Bacteroides*. Collectively, these results suggest that individuals below 75 years of age tend to harbor higher levels of *Actinobacteriota* and Firmicutes-derived genera (*Lachnospirales*, *Bifidobacterium*, and *Blautia*) while older individuals exhibit increased abundance of *Bacteroidota* and *Bacteroides*. This supports the notion of an age-related shift in gut microbial composition, with distinct taxonomic signatures associated with the aging process.

To identify significant differential microbial taxa between age groups, we applied LEfSe, which determines both the statistical significance and the biological relevance of microbial features between groups. The resulting LDA score, expressed on a log scale, quantifies the degree to which a given taxon serves as a potential biomarker differentiating between the groups. LEfSe analysis yielded a set of taxa with significant intergroup differences and meaningful LDA scores, highlighting distinct age-associated microbial signatures (Figure 2). In the <75 age group, the following taxa were identified as significantly enriched: *Actinobacteria* (LDA score = 3.8), *Blautia* (LDA score = 3.5), *Bifidobacterium* (LDA score = 3.2), and *Lachnospirales* (LDA score = 3.0). In contrast, the ≥75 age group was characterized by enrichment in *Bacteroidia* (LDA score = 3.6) and *Bacteroides* (LDA score = 3.4). These findings indicate that younger individuals (aged < 75 years) harbor a gut microbiota enriched in *Actinobacteria* and Firmicutes-related genera such as *Blautia*, *Bifidobacterium*, and *Lachnospirales*. Notably, *Bifidobacterium* and *Blautia* are widely recognized as beneficial gut microbes, and their higher abundance in the younger group may reflect a healthier microbial composition associated with younger age. Conversely, older adults (aged ≥ 75 years) were enriched in *Bacteroidota*-derived taxa, such as *Bacteroidia* and *Bacteroides*, suggesting an age-related microbial shift that may be associated with inflammation or dysbiosis.

### 3.4. Sex-Related Differences in Gut Microbial Community Composition

**Figure 3:**
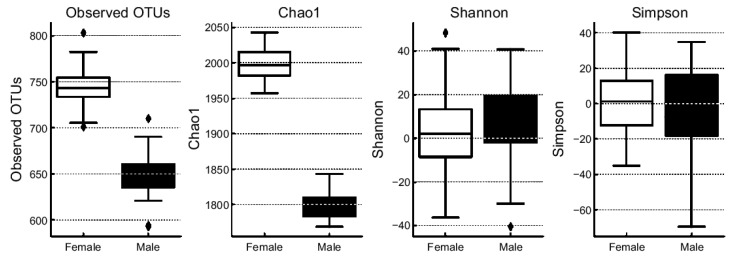
Comparison of alpha diversity metrics (observed OTUs, Chao1, Shannon, and Simpson) between females and males. 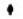 This sign indicates maximum and minimum values.

We analyzed alpha diversity indices to assess differences in gut microbial community structure according to sex (Figure 3). Specifically, we evaluated the observed OTUs, Chao1 richness, Shannon diversity, and Simpson diversity indices to determine how microbial richness, evenness, and overall diversity were distributed between male and female participants. Alpha diversity analysis revealed a generally higher microbial diversity in females than in males (Figure 3). Both the observed OTUs and Chao1 richness indices were significantly higher in females, indicating higher species richness in their gut microbiota. Although Shannon and Simpson indices did not reach statistical significance, females consistently showed higher values for both metrics, suggesting greater microbial evenness and diversity relative to those in males. These findings suggest that females harbor a more diverse and richer gut microbial community than males, highlighting a clear distinction in gut microbiota composition between the sexes.

Taxonomic profiling of the gut microbiota from phylum to species level revealed distinct differences between males and females (Table 5). At the phylum level, Firmicutes was significantly more abundant in females (*p* = 0.01) while *Bacteroidota* was dominant in males (*p* = 0.015). At the class level, females exhibited significantly higher levels of *Actinobacteria* (*p* = 0.002), whereas *Bacteroidia* was relatively more abundant in males (*p* = 0.005). At the order level, *Lachnospirales* was significantly enriched in females (*p* = 0.0008). At the genus level, *Bifidobacterium* was significantly more abundant in females (*p* = 0.003) while *Bacteroides* was more abundant in males (*p* = 0.017). At the species level, females had higher relative abundance of *Blautia* spp., *Faecalibacterium* spp., and *Bifidobacterium* spp. (*p* < 0.005), all of which are generally considered beneficial gut microbes. In contrast, males had higher abundance of *Escherichia coli* (*p* = 0.02) and *Bacteroides* spp. (*p* = 0.03). These results indicate that the composition and diversity of gut microbiota differ meaningfully between sexes, with females tending to harbor a microbiota enriched in beneficial taxa and males exhibiting higher abundance of specific pathobionts and *Bacteroidota*-associated species.

**Figure 4:**
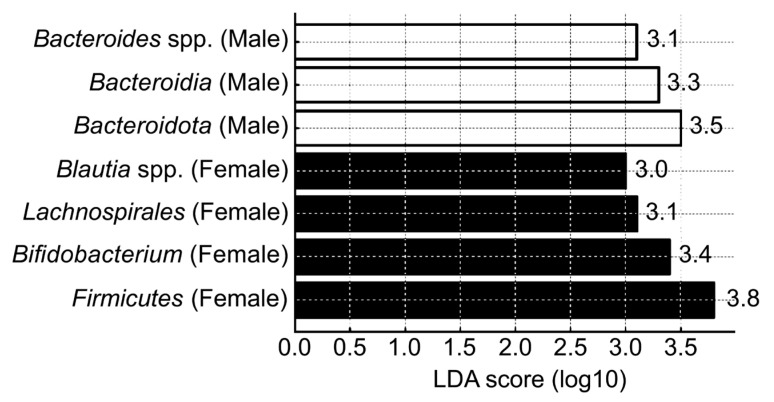
Linear discriminant analysis (LDA) scores showing dominant gut microbiota taxa by sex group (females vs. males).

Using LEfSe, we identified gut microbial taxa that were differentially abundant between males and females, highlighting sex-specific microbial signatures (Figure 4). Several taxa had significantly higher LDA scores in one sex compared with the other, indicating distinct microbial dominance patterns. In the female group, Firmicutes, *Bifidobacterium*, *Lachnospirales*, and *Blautia* spp. exhibited higher LDA scores, suggesting that these taxa were more abundant and relatively enriched in the female gut microbiome. In contrast, the male group showed higher LDA scores for *Bacteroidota*, *Bacteroidia*, and *Bacteroides* spp., indicating dominance of these taxa within the male gut microbiome. Notably, *Blautia* spp. was particularly enriched in females while *Bacteroides* spp. was characteristically enriched in males. The clear separation in dominant taxa between sexes indicate that these microbial features may serve as potential sex-specific biomarkers. Further investigation into the functional roles of these taxa may clarify their relevance in health and disease.

## 4. Discussion

Our findings indicate a marked increase in dementia-related brain changes and pathological burden beginning in the late 60s, with a notable prevalence of mixed pathology (Alzheimer’s + vascular pathology) among individuals aged 70 years and above. The proportion of participants exhibiting moderate to severe brain changes, Alzheimer’s pathology, and vascular pathology each reflecting distinct but often co-occurring mechanisms increased progressively with age (Table 1, Table 2 and Table 3). Alzheimer’s disease is primarily associated with amyloid-beta accumulation and tau pathology, whereas vascular pathology results from cerebrovascular dysfunction, such as ischemic injury or microinfarcts. Although these conditions may interact or co-occur in aging individuals, their biological foundations differ, necessitating separate analysis. Our findings maintain this distinction and independently assess each pathology in relation to gut microbiota composition. Notably, older male participants with vascular pathology were at a significantly higher risk of developing advanced dementia-related brain changes. This suggests that older males with coexisting vascular pathology represent a high-risk group, underscoring the need for multidisciplinary management approaches in dementia care. Preventive strategies aimed at managing vascular risk factors such as hypertension and diabetes may be especially important in this population. Moreover, the results suggest that Alzheimer’s pathology may be more closely linked to subsequent cognitive decline or the onset of dementia symptoms, rather than to the extent of structural brain changes per se (Table 3). This study also provides novel insights into the association between gut microbiota composition and dementia-related brain pathologies across different age and sex groups. In particular, the elderly group (aged ≥ 75 years) exhibited significantly reduced microbial diversity and evident signs of gut dysbiosis, consistent with the findings of previous reports on age-related microbial imbalance. The Shannon and Simpson diversity indices were significantly lower in the elderly group, suggesting a less diverse and less even microbial community. This microbial profile may contribute to a pro-inflammatory gut environment and immunosenescence, which play roles in the pathogenesis of neurodegenerative diseases [26,27,28,29]. In addition, distinct differences in gut microbial composition were identified between age groups. In individuals below the age of 75 years, beneficial microbes belonging to the phyla Firmicutes and *Actinobacteria*, such as *Bifidobacterium* spp., *Blautia* spp., and members of *Lachnospirales*, were predominant. In contrast, individuals aged 75 years and above had a higher relative abundance of *Bacteroidota*, particularly *Bacteroidia* and *Bacteroides* spp. These findings suggests a dominance of pro-inflammatory microbial communities in the older group while the younger group maintains a gut environment enriched in beneficial bacteria [30,31,32,33]. These results are consistent with those of previous reports indicating that aging is associated with an increase in potentially pathogenic gut microbes and a reduction in beneficial taxa. O’Toole and Jeffery [29] reported that aging is accompanied by a decline in Firmicutes, an increase in *Bacteroidetes*, and an overall reduction in microbial diversity [29]. Claesson et al. [34] also demonstrated that with aging, *Bacteroides* spp. increase while beneficial bacteria such as *Bifidobacterium* decrease, directly affecting host health [34]. Biagi et al. [35] further suggested that aging affects the phylogenetic structure of the gut microbiota and its interaction with the immune system. Age-related shifts in microbial communities may contribute to a self-reinforcing loop involving immunosenescence and inflammaging [35]. Additionally, Biagi et al. [35] and Kundu et al. [36] suggested that future research should focus on determining whether administration of the appropriate microbial strains at the right time could improve health outcomes in humans. They emphasized the potential role of gut microbiota as both an indicator and a modifiable determinant of host health [35,36]. In the present study, the female group exhibited a higher proportion of beneficial bacteria belonging to the *Actinobacteria* and Firmicutes phyla whereas the male group exhibited a greater abundance of *Bacteroidota*. These findings suggest that males are more susceptible to developing a pro-inflammatory gut environment while females are more likely to maintain a stable gut ecosystem associated with enhanced immune function.

Although the importance of gut microbial communities to human health has been widely recognized for decades, studies specifically investigating sex-based differences in human gut microbiota remain limited [37,38]. However, animal studies have consistently reported significant sex-related differences in gut microbiota composition (Markle et al. [38,39,40]. Yurkovetskiy et al. [40] reported that the relative abundance of the *Bacteroidaceae* family was higher in male mice than in female mice [40]. Co-occurrence of vascular pathology and pro-inflammatory gut microbiota was associated with a higher likelihood of developing moderate to severe dementia-related brain changes [41]. These findings support the growing body of evidence linking vascular risk factors and gut microbiota to both dementia-related brain pathologies and dementia [42,43]. Our results indicate that gut microbial composition varies distinctly across sex and age, suggesting that microbial profiling provides valuable information for identifying high-risk populations for dementia-related brain pathologies and cognitive impairment. Therefore, future research should focus on developing age- and sex-specific preventive and therapeutic strategies based on gut microbiota profiling. Additionally, mechanistic studies employing multi-omics approaches are needed to elucidate the causal relationships between the gut microbiome and cerebrovascular health. The gut microbiota exhibited distinct structural differences according to age and sex, which were found to be closely associated with the development of dementia-related brain pathologies and cognitive impairment. In particular, beneficial microbes such as *Bifidobacterium* spp. and *Blautia* spp. were more abundant in females below the age of 75 years. These taxa have been reported to exert anti-inflammatory effects, maintain intestinal homeostasis, and produce short-chain fatty acids, all of which contribute to host health [44,45]. Specifically, SCFAs such as butyrate, propionate, and acetate produced by *Bifidobacterium* and *Blautia* modulate immune responses by enhancing regulatory T cell (Treg) differentiation, suppressing pro-inflammatory cytokine production (e.g., IL-6, TNF-α), and inhibiting activation of nuclear factor kappa B (NF-κB) signaling pathways. Furthermore, butyrate strengthens intestinal barrier function by upregulating tight junction proteins, thereby reducing systemic endotoxemia and mitigating neuroinflammation through the gut–brain axis [30,44,45]. In addition, animal studies have suggested their potential roles in improving cognitive function [46]. These findings indicate that such microbial species may serve as protective biomarkers for brain health. In contrast, *Bacteroides* spp. was relatively more abundant in males and individuals aged 75 years and above. Studies have reported associations between *Bacteroides* and pro-inflammatory gut environments, metabolic disorders, and increased risk of cognitive decline and dementia [31,47]. Mechanistically, *Bacteroides* species can promote intestinal inflammation by producing lipopolysaccharides (LPS), which activate toll-like receptor 4 (*TLR4*) signaling pathways, thereby inducing systemic inflammation and contributing to blood–brain barrier dysfunction, neuroinflammation, and ultimately neurodegenerative processes [30,31,47]. Fung et al. [30] also identified altered abundances of *Actinobacteria* and Bacteroidales in patients with Alzheimer’s disease. These results suggest the potential use of *Bacteroides* spp. as risk-monitoring biomarkers, particularly in aging and male populations. Gut microbiota is known to influence brain function and behavior through the microbiota–gut–brain axis [48]. Communication between the gut and brain occurs through the neural, endocrine, and immune pathways [30,49], and accumulating evidence supports the involvement of gut microbiota in neurological disorders [50]. Gut microbes have also been reported to regulate neuroinflammatory pathways via inflammasome signaling [51], and one study found that microbial metabolites can reach the brain and influence gene expression [52]. Interventions using germ-free animals, antibiotics, probiotics, or FMT have also demonstrated the critical role of gut microbes in regulating mood, anxiety, pain, and cognition [49]. Taken together, these findings support a strong interconnection between the gut microbiota, cognitive function, and dementia-related brain pathologies —an association that is further substantiated by the results of the present study. Gut microbiota profiling may serve as an effective tool for the early prediction of dementia-related brain pathologies and cognitive impairment, as well as for the development of personalized prevention strategies. Future research using large-scale cohort studies and multi-omics approaches is warranted to validate these associations and elucidate the underlying mechanisms.

Despite the findings, this study had some limitations. This study had a relatively limited sample size, which may constrain the generalizability of the findings.

Furthermore, as the present study was based on secondary analysis of de-identified biobank data, raw neuroimaging (MRI, PET) and CSF biomarker data were not directly accessible. Although diagnostic classifications were made based on comprehensive clinical assessments at the time of enrollment, the absence of raw diagnostic data limits the ability to present detailed diagnostic findings in this report.

Moreover, the gut microbiome is strongly influenced by external factors such as diet, lifestyle, and medication use, particularly antibiotics, which are confounders that are difficult to fully control for. To obtain more accurate and reproducible results, future studies should consider more stringent control of these environmental and clinical variables.

Future studies should include larger and more diverse populations to enhance the robustness of the analysis. Additionally, we used 16S rRNA gene sequencing to analyze microbial community composition, a method that has inherent limitations in resolving taxa at the species or strain level and in predicting functional and metabolic pathways. Moreover, the gut microbiome is strongly influenced by external factors such as diet, lifestyle, and medication use, particularly antibiotics, which are confounders that are difficult to fully control for. To obtain more accurate and reproducible results, future studies should consider more stringent control of these environmental variables.

## 5. Conclusions

Nonetheless, this study provides meaningful baseline data by characterizing gut microbial differences according to age and sex, and by identifying microbial signatures associated with high-risk groups for dementia-related brain pathologies and cognitive decline. These findings may offer valuable insights for the future prevention and management of neurovascular and neurodegenerative disorders.

## Figures and Tables

**Table 1:** Distribution of dementia-related brain pathologies Alzheimer’s pathology, vascular pathology, and dementia-related structural changes by age and sex (n = 292). Each pathology was evaluated independently to reflect their distinct biological mechanisms.

Age Group (Years)	Sex	Alzheimer’s Pathology n, (%)		Vascular Pathology n, (%)		Dementia-Related Brain Changes n, (%)	
		Positive	Negative	Positive	Negative	Mild	Moderate to Severe
40–59	Male	0 (0.0)	2 (0.7)	1 (0.3)	1 (0.3)	2 (0.7)	0 (0.0)
	Female	3 (1.0)	4 (1.4)	2 (0.7)	5 (1.7)	4 (1.4)	3 (1.0)
60–74	Male	16 (5.5)	38 (13.0)	12 (4.1)	42 (14.4)	11 (3.8)	43 (14.7)
	Female	25 (8.6)	90 (30.8)	26 (8.9)	88 (30.0)	56 (19.5)	58 (19.9)
≥75	Male	13 (4.5)	19 (6.5)	14 (4.8)	18 (6.2)	1 (0.3)	31 (10.6)
	Female	30 (10.3)	52 (17.8)	41 (14.0)	41 (14.0)	20 (6.8)	62 (21.2)
Total		206 (70.5)	86 (29.5)	195 (67.0)	96 (33.0)	94 (32.3)	197 (67.7)
		292 (100)		291 (100)		291 (100)	

Note: Of the 292 participants, 1 did not undergo evaluation for vascular pathology and dementia-related brain changes, resulting in a denominator of 291 for these two categories.

**Table 2:** Multivariate logistic regression predicting risk of Alzheimer’s pathology, vascular pathology, and dementia-related structural brain changes by age and sex.

Outcome	Variable	OR	95% CI (Lower)	95% CI (Upper)	*p*-Value
Alzheimer’s Pathology	Intercept	0.0016	0.00008	0.0349	0.00004
	Age	1.0773	1.0341	1.1223	0.0004
	Sex	1.352	0.7765	2.3541	0.2864
Vascular Pathology	Intercept	0.004	0.0002	0.0712	0.0002
	Age	1.0688	1.028	1.1112	0.0008
	Sex	0.8941	0.5162	1.5486	0.6895
Dementia-Related Brain Changes (Moderate to Severe)	Intercept	0.0015	0.00008	0.0279	0.00001
	Age	1.1008	1.057	1.1464	0.000004
	Sex	4.0575	2.0834	7.9021	0.000038

Note: Sex is coded as male = 1, female = 0; age is a continuous variable. OR, odds ratio; CI: confidence interval.

**Table 3:** Predictors of moderate to severe dementia-related brain changes.

Variable	OR	95% CI (Lower)	95% CI (Upper)	*p*-Value
Intercept	0.002380	0.000113	0.049988	0.000101
Sex	4.239421	2.155785	8.336959	2.836338
Age	1.086813	1.041521	1.134075	0.000127
Alzheimer’s Pathology (Positive)	1.385263	0.732585	2.619428	0.316046
Vascular Pathology (Positive)	3.555244	1.833334	6.894412	0.000174

**Table 4:** Taxonomic composition showing significant differences in dominant taxa between age groups (<75 vs. ≥75 years).

Taxonomic Level	Taxon	Dominant Group (Age)	*p*-Value
Phylum	*Actinobacteriota*	<75	0.004
	*Bacteroidota*	≥75	0.005
Class	*Actinobacteria*	<75	0.0005
	*Bacteroidia*	≥75	0.005
Order	*Lachnospirales*	<75	0.00006
Genus	*Bifidobacterium*	<75	0.001
	*Bacteroides*	≥75	0.0179
Species	*Blautia* spp.	<75	0.001
	*Faecalibacterium* spp.	<75	0.003
	*Bifidobacterium* spp.	<75	0.005
	*Escherichia coli*	≥75	0.015
	*Bacteroides* spp.	≥75	0.02

Note: Dominant Group, age group with a higher relative abundance of the corresponding microbial taxa.; *p*-value, statistical significance based on the Wilcoxon rank-sum test.

**Table 5:** Taxonomic composition showing significant differences in dominant taxa between sexes.

Taxonomic Level	Taxon	Dominant Group	*p*-Value
Phylum	Firmicutes	Female	0.01
	*Bacteroidota*	Male	0.015
Class	*Actinobacteria*	Female	0.002
	*Bacteroidia*	Male	0.005
Order	*Lachnospirales*	Female	0.0008
Genus	*Bifidobacterium*	Female	0.003
	*Bacteroides*	Male	0.017
Species	*Blautia* spp.	Female	0.002
	*Faecalibacterium* spp.		0.003
	*Bifidobacterium* spp.		0.005
	*Escherichia coli*	Male	0.02
	*Bacteroides* spp.		0.03

Note: Dominant Group, sex group with a higher relative abundance of the corresponding microbial taxa; *p*-value, statistical significance based on the Wilcoxon rank-sum test.

## Data Availability

The datasets generated and/or analyzed during the current study are not publicly available owing to the inclusion of sensitive patient information, although are available from the corresponding author on reasonable request.

## Abbreviations

The following abbreviations are used in this manuscript:

BICWALZS	Biobank Innovations for Chronic Cerebrovascular Disease with Alzheimer’s Disease Study
EV	Extracellular vesicles
FMT	Fecal microbiota transplantation
LDA	Linear discriminant analysis
LEfSe	LDA effect size
MRI	Magnetic resonance imaging
OR	Odds ratio
OTUs	Operational taxonomic units
PET	Positron emission tomography
